# Mycoplasma pneumoniae among Chinese Outpatient Children with Mild Respiratory Tract Infections during the Coronavirus Disease 2019 Pandemic

**DOI:** 10.1128/spectrum.01550-21

**Published:** 2022-02-09

**Authors:** Jiande Chen, Jing Zhang, Zhiwei Lu, Yu Chen, Songsong Huang, Hengtao Li, Shuzhu Lin, Jun Yu, Xueqi Zeng, Cuihong Ji, Yuejie Zheng, Fangfang Dai, Wei Dong, Huiting Xu, Weichao Chen, Xiaoqun Jin, Zhen Cui, Jing Qiao, Wei Qin, Hui Chen, Wei Jiang, Xiaoying Zhang, Jingrong Song, Jie Shao, Wen Su, Chao Wang, Fang Liu, Yuhua Zhao, Yingxue Zou, Run Guo, Lei Zhang, Jinhong Wu, Shuhua Yuan, Mingyu Tang, Yufen Wu, Jie Lin, Wenfang Dong, Xing Chen, Xinrong Sun, Yong Yin

**Affiliations:** a Department of Respiratory Medicine, Shanghai Children’s Medical Center, School of Medicine, Shanghai Jiao Tong University, Shanghai, China; b Department of Respiratory Diseases, Shenzhen Children’s Hospital, Shenzhen, Guangdong, China; c Department of Pediatrics, Shanghai Fourth People’s Hospital, Tongji University, Shanghai, China; d Department of Pediatrics, Fengcheng Hospital, Shanghai, China; e Department of Pediatrics, Fengxian District Central Hospital, Shanghai, China; f Department of Pediatrics, Shandong Provincial Hospital Affiliated to Shandong First Medical University, Jinan, Shandong, China; g Department of Pediatrics, Nanxiang Hospital, Shanghai, China; h Second Department of Infectious Diseases, Xi’an Children’s Hospital, The Affiliated Children’s Hospital of Xi’an Jiaotong University, Shaanxi, China; i Department of Pediatrics, Putuo District People’s Hospital, Shanghai, China; j Department of Pediatrics, Shanghai East Hospital, Tongji University School of Medicine, Shanghai, China; k Department of Pediatrics, Dahua Hospital of Shanghai Xuhui District, Shanghai, China; l Department of Pediatrics, Shanghai Ninth People’s Hospital, Shanghai Jiao Tong University School of Medicine, Shanghai, China; m Department of Pediatrics, Ruijin Hospital, Shanghai Jiao Tong University School of Medicine, Shanghai, China; n Department of Pulmonary, Shanghai Children’s Hospital, Shanghai Jiao Tong University, Shanghai, China; o Department of Respiratory Medicine, Tianjin Children’s Hospital (Children’s Hospital of Tianjin University), Tianjin, China; University of Mississippi Medical Center

**Keywords:** *Mycoplasma pneumoniae*, children, macrolide, outpatient, COVID-19

## Abstract

Mycoplasma pneumoniae is a common pathogen causing respiratory disease in children. We sought to investigate the epidemiology of M. pneumoniae among outpatient children with mild respiratory tract infections (RTIs) during the coronavirus disease 2019 (COVID-19) pandemic. Eligible patients were prospectively enrolled from January 2020 to June 2021. Throat swabs were tested for M. pneumoniae RNA. M. pneumoniae IgM was tested by a colloidal gold assay. Macrolide resistance and the effect of the COVID-19 countermeasures on M. pneumoniae prevalence were assessed. Symptom scores, treatments, and outcomes were evaluated. Eight hundred sixty-two eligible children at 15 centers in China were enrolled. M. pneumoniae was detected in 78 (9.0%) patients. Seasonally, M. pneumoniae peaked in the first spring and dropped dramatically to extremely low levels over time until the next summer. Decreases in COVID-19 prevalence were significantly associated with decreases in M. pneumoniae prevalence (*r *=* *0.76, *P = *0.001). The macrolide resistance rate was 7.7%. The overall sensitivity and specificity of the colloidal gold assay used in determining M. pneumoniae infection were 32.1% and 77.9%, respectively. No more benefits for improving the severity of symptoms and outcomes were observed in M. pneumoniae-infected patients treated with a macrolide than in those not treated with a macrolide during follow-up. The prevalences of M. pneumoniae and macrolide resistance in outpatient children with mild RTIs were at low levels in the early stage of the COVID-19 pandemic but may have rebounded recently. The colloidal gold assay for M. pneumoniae IgM may be not appropriate for diagnosis of M. pneumoniae infection. Macrolides should be used with caution among outpatients with mild RTIs.

**IMPORTANCE** This is the first and largest prospective, multicenter, active, population-based surveillance study of the epidemiology of Mycoplasma pneumoniae among outpatient children with mild respiratory tract infections (RTIs) during the COVID-19 pandemic. Nationwide measures like strict face mask wearing and restrictions on population movement implemented to prevent the spread of COVID-19 might also effectively prevent the spread of M. pneumoniae. The prevalence of M. pneumoniae and the proportion of drug-resistant M. pneumoniae isolates in outpatient children with mild RTIs were at low levels in the early stage of the COVID-19 pandemic but may have rebounded recently. The colloidal gold assay for M. pneumoniae IgM may be not appropriate for screening and diagnosis of M. pneumoniae infection. Macrolides should be used with caution among outpatients with mild RTIs.

## INTRODUCTION

Mycoplasma pneumoniae is a common pathogen among children with upper or lower respiratory tract infections (RTIs) (1–3). Together with a high prevalence of M. pneumoniae infections, a high prevalence of macrolide resistance in M. pneumoniae strains was also observed among Chinese pediatric patients with RTIs (2). However, during the pandemic of coronavirus disease 2019 (COVID-19), which is caused by severe acute respiratory syndrome coronavirus 2 (SARS-CoV-2), pneumonia (4) and hospitalized RTI cases (3) caused by M. pneumoniae seemed to decrease rapidly. Under the circumstance of the ongoing pandemic of COVID-19, the epidemiology of M. pneumoniae among outpatient children with mild RTIs is unknown.

Detection of M. pneumoniae can be achieved by culture, serology, or molecular-based methods (5). Because of the greater analytical sensitivity and specificity and shorter turnaround time of nucleic acid amplification tests, they have been considered the new “gold standard” of M. pneumoniae infections (6). Recently, the colloidal gold immunochromatographic assay for diagnosing M. pneumoniae infection has also seemed to be a rapid, sensitive, and specific method (7, 8).

The emergence of macrolide-resistant strains has become widespread all over the world (1, 9–11), especially in Asia. In China, the rate of macrolide-resistant M. pneumoniae isolates has been assessed to be over 50% (12). However, the rate of macrolide-resistant M. pneumoniae isolates among Chinese outpatient children with mild RTIs during the COVID-19 pandemic is unknown.

With low MICs against the bacterium, low toxicity, and the absence of contraindications in young children, macrolides are a first-line treatment for M. pneumoniae RTIs (13). However, the majority of M. pneumoniae infections have mild and self-limited symptoms, and severe courses of disease are not common (5, 14, 15). Excessive or inappropriate use of macrolides may lead to a selective pressure for the development of macrolide resistance (16).

This study was a prospective, multicenter, active, population-based surveillance study of the epidemiology of M. pneumoniae among outpatient children with mild RTIs during the COVID-19 pandemic. The diagnostic capacity of a colloidal gold assay for M. pneumoniae infection and the effects of a macrolide on mild RTIs were also determined.

## RESULTS

### Study participants.

Eight hundred sixty-two eligible children were enrolled. The characteristics of the children included are listed in Table 1. The median age was 4 years (interquartile range [IQR], 2 to 6 years), 44.3% were female, 17.6% had a macrolide prescription within 1 month prior to study enrollment, and 26.9% were prescribed a macrolide (azithromycin) on day 0.

**TABLE 1:** Participant characteristics

Characteristic	Value [no. (%) or median (IQR)] for patients with indicated M. pneumoniae RNA result diagnosed with^*a*^:							
	Mild respiratory tract infection (*n* = 862)		Common cold (*n* = 393)		Acute bronchitis (*n* = 416)		Postinfection cough (*n* = 53)	
	Positive (*n* = 78)	Negative (*n* = 784)	Positive (*n* = 33)	Negative (*n* = 360)	Positive (*n* = 41)	Negative (*n* = 375)	Positive (*n* = 4)	Negative (*n* = 49)
Age (yr)								
<2	21 (26.9)	180 (23.0)	12 (36.4)	93 (25.8)	9 (22.0)	77 (20.5)	0 (0.0)	10 (20.4)
2–4	26 (33.3)	292 (37.2)	10 (30.3)	115 (31.9)	16 (39.0)	146 (38.9)	0 (0.0)	31 (63.3)
5–9	25 (32.1)	253 (32.3)	10 (30.3)	123 (34.2)	12 (29.3)	122 (32.5)	3 (75.0)	8 (16.3)
10–18	6 (7.7)	59 (7.5)	1 (3.0)	29 (8.1)	4 (9.8)	30 (8.0)	1 (25.0)	0 (0.0)
Male	51 (65.4)	429 (54.7)	19 (57.6)	188 (52.2)	28 (68.3)	216 (57.6)	4 (100.0)	25 (51.0)
Clinical presentation								
Fever/feverish	42 (53.8)	324 (41.3)	22 (66.7)	214 (59.4)	20 (48.8)	110 (29.3)	0 (0.0)	0 (0.0)
Severity of symptoms^*b*^	1.0 (0.8–1.4)	1.0 (0.6–1.4)	1.0 (0.6–1.3)	0.8 (0.5–1.3)	1.0 (0.8–1.4)	1.1 (0.8–1.4)	1.0 (0.7–1.2)	0.9 (0.6–1.1)
Duration of symptoms before visit (days)	4 (2–7)	3 (2–7)	3 (2–3)	2 (1–3)	5 (3–7)	4 (2–8)	45 (30–68)	30 (30–30)
Laboratory findings								
M. pneumoniae IgM positive	25 (32.1)	173 (22.1)	5 (15.2)	66 (18.3)	18 (43.9)	99 (26.4)	2 (50.0)	8 (16.3)
C-reactive protein level (mg/L)	4.3 (0.5–10.0)	1.6 (0.5–5.2)	4.3 (0.5–7.6)	2.7 (0.6–8.3)	5 (0.5–11.9)	1.0 (0.5–4.0)	0.5 (0.4–0.6)	0.6 (0.5–1.7)
Leukocyte count (×10^9/L)	7.7 (5.7–10.8)	8.1 (6.4–10.2)	8.1 (5.5–11.7)	8.1 (6.3–10.6)	7.5 (6.2–10.1)	8.0 (6.6–10.0)	7.7 (6.4–9.8)	8.0 (6.6–9.8)
Receipt of antibiotic								
Macrolide during 1 mo prior to study enrollment	17 (21.8)	135 (17.2)	3 (9.1)	29 (8.1)	14 (34.1)	91 (24.3)	1 (25.0)	15 (30.6)
Macrolide (azithromycin) on day 0	28 (35.9)	204 (26.0)	6 (18.2)	53 (14.7)	20 (48.8)	140 (37.3)	2 (50.0)	11 (22.4)

a IQR, interquartile range.

b The severity of symptoms was defined as the symptom score of each patient divided by the reference value for each subgroup. The reference values, which were the median symptom scores of patients with Mycoplasma pneumoniae RNA positivity on day 0, were 10, 8.5, and 9.8 for the common cold, acute tracheobronchitis, and postinfection cough subgroups, respectively.

### M. pneumoniae nucleic acid detection.

**FIG 1:**
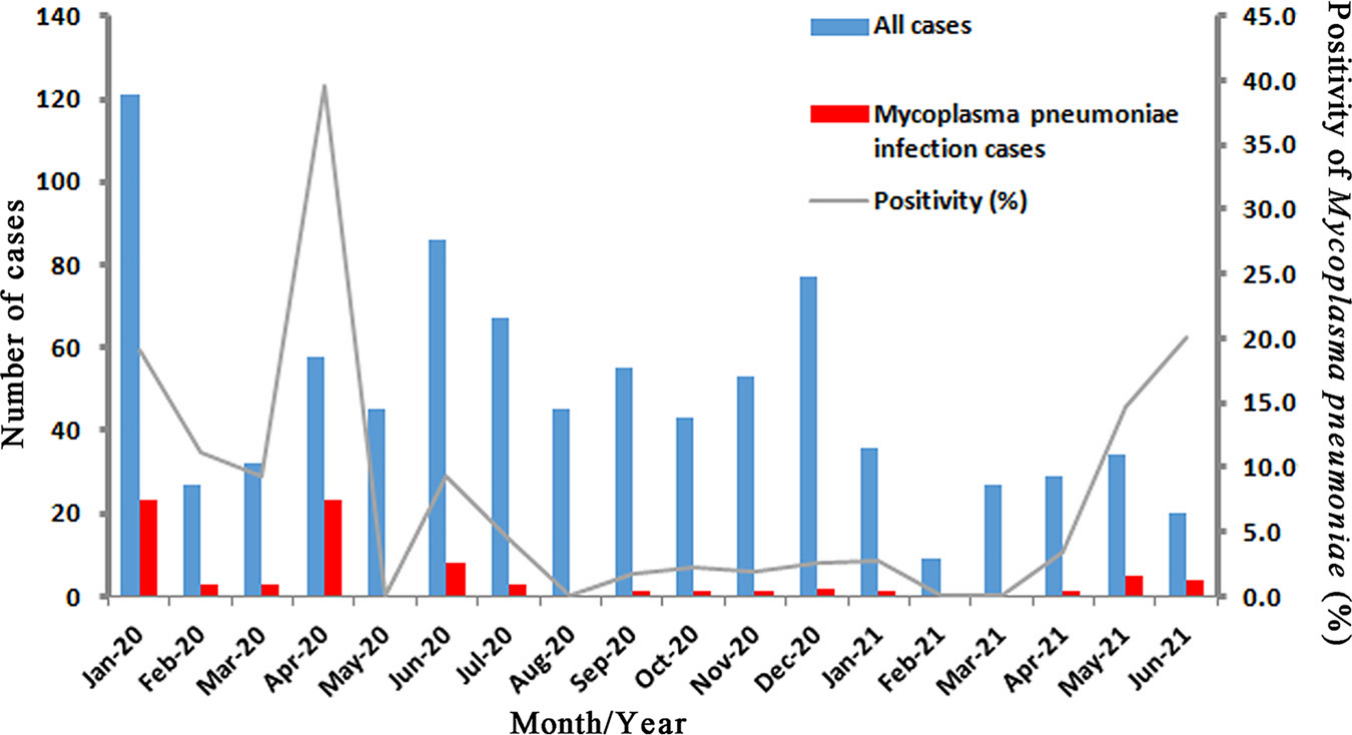
Monthly cases and positivity of Mycoplasma pneumoniae from January 2020 to June 2021 in the COVID-19 pandemic.

Among 862 outpatient children with mild RTIs, M. pneumoniae was detected in 78 (9.0%) by RNA isothermal amplification-gold probe chromatography. Mutations associated with resistance to macrolides were detected in isolates from 6 (7.7%) patients with M. pneumoniae infection. Only the A2063G mutation (a change of A to G at position 2063) was found (100%). Seasonally, M. pneumoniae peaked in the first spring and dropped dramatically to extremely low levels over time until the next summer (Fig. 1).

### Effect of the COVID-19 countermeasures on M. pneumoniae prevalence.

**FIG 2:**
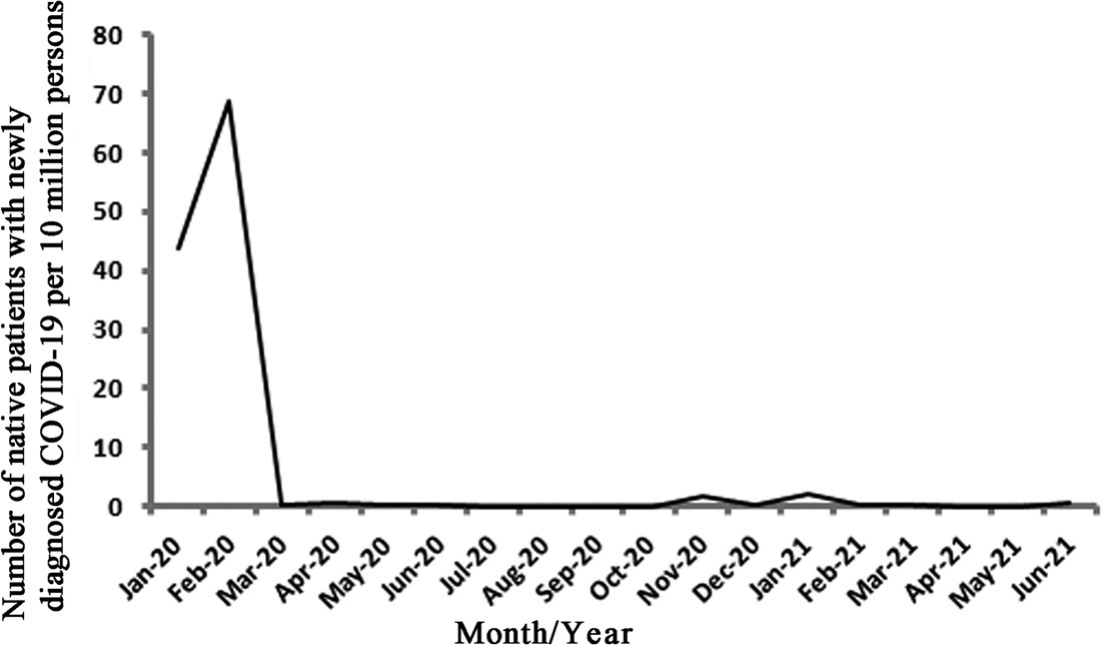
Number of native newly diagnosed COVID-19 patients per 10 million persons per month from January 2020 to June 2021.

The number of native newly diagnosed COVID-19 patients per 10 million persons per month is shown in Fig. 2. Similar changes in the patterns of prevalence of COVID-19 and M. pneumoniae could be seen in the early stage of the COVID-19 pandemic. Compared with the curve for M. pneumoniae (Fig. 1), the peak value of the curve for COVID-19 appeared 2 months earlier. Considering the response lag in M. pneumoniae prevalence following the initiation of COVID-19 countermeasures, the prevalence data for M. pneumoniae from March 2020 to June 2021 and the prevalence data of COVID-19 from January 2020 to April 2021 were used to perform Pearson correlation coefficient analysis. Decreases in COVID-19 prevalence were significantly associated with decreases in M. pneumoniae prevalence (*r *=* *0.76, *P = *0.001). When the prevalence data of COVID-19 from January 2020 to July 2020 and the prevalence data of M. pneumoniae from March 2020 to September 2020 were used to perform Pearson correlation coefficient analysis, the value of *r* was 0.88 (*P = *0.009). This showed that the COVID-19 countermeasures might have had a stronger impact on reducing the prevalence of M. pneumoniae in the early stage of the COVID-19 pandemic.

### Discriminatory capacity of colloidal gold assay.

Analysis of the results of M. pneumoniae RNA detection by RNA isothermal amplification-gold probe chromatography showed that the overall sensitivity and specificity, positive and negative predictive values, and positive and negative likelihood ratios of the colloidal gold assay used in differentiating presence from absence of M. pneumoniae infection were 32.1%, 77.9%, 12.6%, 92.0%, 1.45, and 0.87, respectively. The proportion of macrolide misuse driven by positive results of the colloidal gold assay was 89.4%. To include the sampling time point after symptoms in the analysis and reflect what actually occurs in clinical practice at the same time, we further analyzed the discriminatory capacity of the colloidal gold assay among the three patient subgroups (patients diagnosed with the common cold, acute tracheobronchitis, and postinfection cough [PIC]) (Table 2). Although the discriminatory capacity of the colloidal gold assay improved with a delay in the detection time point, the improvement was not good enough. The maximum sensitivity and specificity were only 50.0% and 83.7%, respectively.

**TABLE 2:** Discriminatory capacity of the colloidal gold assay for M. pneumoniae infection

Parameter	Value [% (95% CI) unless otherwise indicated] for patients diagnosed with^*a*^:			
	Mild respiratory tract infection (*n* = 862)	Common cold (*n* = 393)	Acute bronchitis (*n* = 416)	Postinfection cough (*n* = 53)
Sampling time point after symptoms (days) [median (IQR)]	3 (2–7)	2 (1–3)^*b*^	4 (3–8)^*b*^	30 (30–30)^*b*^
Sensitivity	32.1 (21.9–43.6)	15.2 (5.1–31.9)	43.9 (28.5–60.3)	50.0 (6.8–93.2)
Specificity	77.9 (74.9–80.8)	81.7 (77.3–85.5)	73.6 (68.8–78.0)	83.7 (70.3–92.7)
Positive predictive value	12.6 (8.3–18.1)	7.0 (2.3–15.7)	15.4 (9.4–23.2)	20.0 (2.5–55.6)
Negative predictive value	92.0 (89.7–94.0)	91.3 (87.7–94.1)	92.3 (88.7–95.1)	95.3 (84.2–99.4)
Positive likelihood ratio (95% CI)	1.45 (1.02–2.06)	0.83 (0.36–1.91)	1.66 (1.13–2.44)	3.06 (0.95–9.84)
Negative likelihood ratio (95% CI)	0.87 (0.75–1.02)	1.04 (0.89–1.21)	0.76 (0.58–1.01)	0.60 (0.22–1.60)

a CI, confidence interval.

b The sampling time points after symptoms were significantly different among the three subgroups (comparisons between any two groups were performed by Mann-Whitney *U* test, and all *P* values were <0.001).

### Effect of macrolide on outpatient children with M. pneumoniae infection.

**FIG 3:**
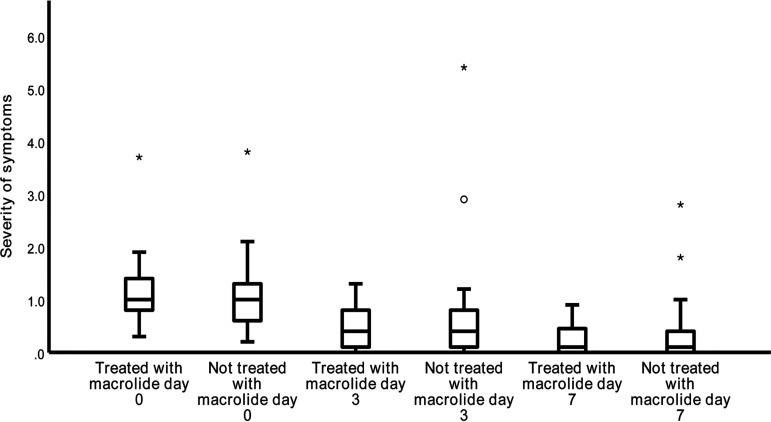
Comparison of the severity of symptoms between the group of M. pneumoniae-infected patients treated with a macrolide and the group of M. pneumoniae-infected patients not treated with a macrolide at days 0, 3, and 7. The severity of symptoms was defined as the symptom score of each patient divided by the reference value. The reference values, which were the median symptom scores of patients with Mycoplasma pneumoniae RNA positivity on day 0, were 10, 8.5, and 9.8 for the common cold, acute tracheobronchitis, and postinfectioncough subgroups, respectively. Asterisks and the open circle represent outliers.

M. pneumoniae infection patients (*n* = 78) were stratified by whether a macrolide (azithromycin) was used (*n* = 28) or not (*n* = 50) at the initial visit. There was no significant difference in the severity of symptoms between the group of M. pneumoniae-infected patients treated with the macrolide and the group of M. pneumoniae-infected patients not treated with the macrolide at days 0, 3, and 7 (Fig. 3). During the follow-up period, no significantly lower frequency of repeat outpatient visits, pneumonia development, or hospitalization was observed in the group of M. pneumoniae-infected patients treated with the macrolide compared with these parameters in the group of M. pneumoniae-infected patients not treated with the macrolide (Table 3).

**TABLE 3:** Follow-up of patients with Mycoplasma pneumoniae infection

Parameter	No. (%) of M. pneumoniae-infected patients who were:		*P* value
	Treated with macrolide (*n* = 28)	Not treated with macrolide (*n* = 50)	
Repeat outpatient visits	4 (14.3)	3 (6.0)	0.243
Pneumonia development	2 (7.1)	2 (4.0)	0.615
Hospitalization	3 (10.7)	0 (0.0)	0.043

## DISCUSSION

To our knowledge, this is the first and largest prospective, multicenter, active, population-based surveillance study of the epidemiology of M. pneumoniae among outpatient children with mild RTIs during the COVID-19 pandemic. A dramatic decrease in the M. pneumoniae infection rate among these patients was noticed in the early stage of the COVID-19 pandemic (Fig. 1).

The natural epidemic cycle of M. pneumoniae could not explain this phenomenon. First, a national multicenter prospective surveillance of all-age patients (52.2% were aged <18 years) with acute RTIs conducted in China between 2009 and 2019 showed a noticeable peak in the positivity rate of M. pneumoniae in 2011 and a gradual upward trend of the positivity rates of M. pneumoniae from 2015 to 2019 (most of the positive M. pneumoniae results were detected in pediatric patients) (17). Therefore, the prevalence of M. pneumoniae in pediatric patients with RTIs in 2020 was expected to be another peak, not a decline. Second, the facts that the M. pneumoniae infection rate decreased dramatically from the peak to an extremely low level within 1 month and that the extremely low level lasted for almost 1 year could not be explained by normal seasonal trends. No similar seasonal distribution of positivity rates of M. pneumoniae among pediatric patients with RTIs could be seen in previous studies in China (3, 18, 19). Third, M. pneumoniae infections tend to peak during summer or early fall (3, 5, 18, 19). However, the positivity rate of M. pneumoniae was extremely low in this period of 2020 in our study.

In China, extraordinary public health measures have been implemented since the COVID-19 outbreak (20). COVID-19 has been included in B class infectious diseases according to the law of the People’s Republic of China on the prevention and treatment of infectious diseases and has been managed as an A class infectious disease since 20 January 2020 (21). A dramatic downward turn of the COVID-19 epidemic curve (Fig. 2) suggested that the COVID-19 countermeasures effectively curbed the pandemic. Like SARS-CoV-2, M. pneumoniae bacteria spread from person-to-person contact by respiratory droplets created by the coughing and sneezing of someone infected with M. pneumoniae. Therefore, the implementation of nationwide countermeasures like strict face mask wearing and restrictions on population movement to prevent the spread of COVID-19 might also have effectively prevented the spread of M. pneumoniae and led to the dramatic decrease in the M. pneumoniae infection rate in the early stage of the COVID-19 pandemic and the low overall M. pneumoniae prevalence in our study. To support this hypothesis, we indirectly proved the effect of the COVID-19 countermeasures on the decreases in M. pneumoniae prevalence by exploring the relationship between the prevalence of M. pneumoniae and the prevalence of COVID-19. Decreases in COVID-19 prevalence were significantly associated with decreases in M. pneumoniae prevalence (*r *=* *0.76, *P = *0.001), especially in the early stage of the COVID-19 pandemic (*r *=* *0.88, *P = *0.009). Our study, however, was observational and limited to pediatric outpatients with mild RTIs. Thus, high-quality randomized trials to definitively address the impact of the COVID-19 countermeasures on the overall prevalence of M. pneumoniae may be needed.

In the control of the COVID-19 pandemic, for the general public, it is not necessary to wear masks at home and outdoors when there is good ventilation and no crowds (22). Active and orderly resumption of work and production and convenient and orderly flow of personnel were proposed by the government on 7 April 2020 (23). On 13 May 2020, the reopening of schools and classes at all levels in education was promoted by the Ministry of Education of the People’s Republic of China (24). These measures, as well as the arrival of summer, might be the reasons for the recent upward trend of the positivity rate of M. pneumoniae (Fig. 1).

The prevalence of macrolide resistance in M. pneumoniae strains among Chinese children with RTIs has been considered to be very high based on previous epidemiological studies (2, 25). However, in our study, the prevalence of macrolide resistance of M. pneumoniae strains among outpatient children with mild RTIs was 7.7%, which is very similar to the levels of prevalence in the United States (1) and France (26). Possible reasons for this decrease in prevalence could be as follows: (i) in terms of population distribution, the macrolide resistance rates of M. pneumoniae strains may tend to be lower among outpatients (27) or patients with mild RTIs (2) than in inpatients or patients with pneumonia, and (ii) the macrolide resistance of M. pneumoniae develops *de novo* via replication during macrolide therapy (5, 28) and selective pressure for the development of antibiotic resistance may be lower among outpatients with mild RTIs than in inpatients. Only 17.6% of the outpatients with mild RTIs had prior macrolide prescriptions in our study, while macrolide usage prior to hospital admission was estimated to be over 80% in hospitalized children with M. pneumoniae pneumonia (29).

Similar to other studies (30, 31), poor overall sensitivity and specificity of 32.1% and 77.9%, respectively, for M. pneumoniae IgM as measured by the colloidal gold assay were observed in our study. Subgroup analysis showed that its discriminatory capacity improved with a delay in the detection time point (Table 2). However, the improvement was not good enough. The maximum sensitivity and specificity were only 50.0% and 83.7%, respectively. The use of samples taken in the second week after disease onset may improve its discriminatory capacity, but this may lack practical significance in application (5). In China, most pediatric patients with a common cold or acute tracheobronchitis may go to see a doctor and be tested for M. pneumoniae within 1 week after disease onset rather than in the second week. Patients with RTIs do not meet the diagnostic criteria for postinfection cough (PIC) in the second week, and patients with PIC may tend to choose a more accurate pathogen detection method, such as a nucleic acid amplification test, because of long-term cough. Not only might the results of serological tests miss persons who are infected, but their nonspecific performance will also result in false positives (5). The proportion of macrolide misuse guided by the positive results obtained using colloidal gold in our study was 89.4%. Therefore, the colloidal gold assay for M. pneumoniae IgM might not be effective for a rapid diagnosis of M. pneumoniae infection among outpatient children with mild RTIs.

Macrolides are generally considered to be first-line antibiotics for M. pneumoniae infections. However, in our study, the use of a macrolide did not improve the severity of symptoms (Fig. 3) and the outcomes (Table 3) of the outpatients with M. pneumoniae infection compared with the severity of symptoms and outcomes for the group of M. pneumoniae-positive outpatients without macrolide treatment. Overuse of macrolides could lead to the emergence of macrolide-resistant M. pneumoniae strains with time (32). In addition, overuse of macrolides can give rise to side effects like prolongation of the QT interval (33). Therefore, macrolides should be used with caution to treat suspected M. pneumoniae infection in outpatient children with mild RTIs. Macrolides may not benefit these patients, but further studies with larger samples are needed to confirm it.

Our study has several limitations. First, terminating our study at the time when the M. pneumoniae infection rate was in an upward trend resulted in lack of knowledge of subsequent changes. Second, we included patients with mild RTIs rather than all patients with RTIs, so the overall situation of M. pneumoniae infections in outpatients could not be observed. Third, we did not carry out the detection of other common pathogens or molecular typing of M. pneumoniae, so whether there were coinfections and the prevalence of *p1* genotypes are unknown.

In conclusion, the prevalence of M. pneumoniae and the proportion of drug-resistant M. pneumoniae strains in outpatient children with mild RTIs were at low levels in the early stage of the COVID-19 pandemic but may have rebounded recently. The decrease in the prevalence of M. pneumoniae might be due to the COVID-19 countermeasures. The colloidal gold assay for M. pneumoniae IgM may not be appropriate for screening and diagnosis of M. pneumoniae infection among outpatient children with mild RTIs. Macrolides should be used with caution among outpatients with mild RTIs.

## MATERIALS AND METHODS

### Participants and setting.

From January 2020 to June 2021, children aged 28 days to 18 years who attended Shanghai Children’s Medical Center and 14 other hospitals in China with doctor-diagnosed mild RTIs (common cold, acute tracheobronchitis, or postinfection cough) were screened for eligibility. The common cold was diagnosed according to the Chinese expert consensus on standardized diagnosis and treatment of common cold in children (2013) (34), as follows: (i) acute onset; (ii) symptoms of nasal and pharyngeal catarrh, such as sneezing, nasal stuffiness and discharge, sore throat, and cough; (iii) fever may be present or not; and (iv) exclusion of other diseases. Acute tracheobronchitis was diagnosed according to the guidelines for primary care of acute tracheobronchitis, practice version (2018) (35), as follows: (i) acute onset; (ii) cough as the main symptom; (iii) the presence of at least one other respiratory symptom, such as expectoration, wheeze, or chest pain; and (iv) exclusion of other diseases. Postinfection cough (PIC) was diagnosed according to the guidelines for diagnosis and treatment of chronic cough in Chinese children (2013) (36), as follows: (i) recent history of respiratory tract infection; (ii) cough for >4 weeks, manifested as irritative dry cough or with a little white mucus; (iii) no abnormality shown by chest X-ray; (iv) normal result in pulmonary function test; and (v) exclusion of other diseases.

Participants who had underlying disease(s) like congenital heart disease, immunodeficiency, and hereditary disease, were allergic to macrolides, had poor compliance (defined as inability to fill in the questionnaires on time and truthfully), or did not simultaneously carry out an M. pneumoniae colloidal gold assay of a blood sample and M. pneumoniae nucleic acid detection by throat swab sample were excluded.

This was a prospective multicenter surveillance study. Written consent was obtained from the parents of all participants. The study was conducted in accordance with current good clinical practice, and the protocol was approved by an Independent Ethics Committee or Institutional Review Board for each center (no. SCMCIRB-K2019060-1).

### Sample size.

Two-sided confidence intervals (CIs) for one proportion (37, 38) were used for sample size calculation by PASS 12 Power Analysis and Sample Size software (NCSS, LLC, Kaysville, UT, USA). A sample size of 715 produces a two-sided 95% confidence interval with a width equal to 0.060 when the sample proportion is 0.2 (18). Because this was a prevalence study, cases continued to be included after reaching the target sample size.

### Study protocol.

The first visit was set as day 0. Throat swab specimens were collected for M. pneumoniae RNA detection and DNA sequencing at day 0. SARS-CoV-2 detection was not performed unless COVID-19 was suspected. Complete blood count, a blood test for C-reactive protein, and colloidal gold assay for M. pneumoniae IgM detection were also performed at the first visit. On day 0, through free instant messaging software (WeChat; Tencent, Shenzhen, China) available on smart phones, parents would receive questionnaires (Table S1 in the supplemental material for common cold patients and Table S2 for acute tracheobronchitis and PIC patients) and be asked to complete them truthfully. Besides the degree of symptoms (Table S1 or S2), information about the frequency of repeat outpatient visits, development of pneumonia, and hospitalization (Table S3) for each patient was also collected on day 3 and day 7. To normalize the degree of symptoms, the median symptom score of patients with M. pneumoniae RNA positivity on day 0 was calculated as the reference value for each subgroup. The severity of symptoms was defined as the symptom score of each patient divided by the reference value.

If the attending pediatrician considered using a macrolide to treat a patient with suspected M. pneumoniae infection, only azithromycin could be used. The dosage of azithromycin was 10 mg/kg of body weight once daily for three consecutive days. The proportion of macrolide misuse driven by positive results from the colloidal gold test was calculated as follows: number of cases of macrolide misuse/(number of cases where macrolide use was correct + number of cases of misuse).

### Effect of the COVID-19 countermeasures on M. pneumoniae prevalence.

The prevalence of COVID-19 is directly related to the COVID-19 countermeasures. Therefore, we studied the correlation between the prevalence of COVID-19 and the prevalence of M. pneumoniae to explore the effect of the COVID-19 countermeasures on M. pneumoniae prevalence. For each center, the number of native patients with newly diagnosed COVID-19 per month in the city or province where the center is located was obtained from the website of the government’s Health Commission (https://wsjkw.sh.gov.cn/, http://wjw.sz.gov.cn/, http://jnmhc.jinan.gov.cn/, http://sxwjw.shaanxi.gov.cn/, http://wsjk.tj.gov.cn/). The size of the resident population of the city or province where the center is located was obtained from the website of the government’s Bureau of Statistics (https://tjj.sh.gov.cn/, http://tjj.sz.gov.cn/, http://jntj.jinan.gov.cn/, http://tjj.shaanxi.gov.cn/, http://stats.tj.gov.cn/). Based on these data, the number of native patients with newly diagnosed COVID-19 per 10 million persons per month was calculated.

### Colloidal gold assay for M. pneumoniae IgM detection.

The qualitative determination of M. pneumoniae IgM antibodies that may be present in human whole blood was detected using a Mycoplasma pneumoniae IgM antibody test kit (colloidal gold method) (Wiz Biotech, Xiamen, China) according to the manufacturer’s instructions. Briefly, 20 μl of fingertip blood together with 100 μl of diluent was added into the sample well for 10 to 15 min. Samples with red bands in both the control area and the test area were considered M. pneumoniae IgM positive.

### RNA isothermal amplification-gold probe chromatography for M. pneumoniae RNA detection.

The qualitative determination of M. pneumoniae RNA that may be present in a throat swab sample was performed using a Mycoplasma pneumoniae nucleic acid detection kit (RNA isothermal amplification-gold probe chromatography) (Wuhan Zhongzhi Biotechnologies, Inc., China). Briefly, a collected sample was treated by cell lysis solutions to release pathogen nucleic acids. The pathogen nucleic acids released were amplified by reverse transcriptase and T7 RNA polymerase. The amplified products were recognized and captured by specific probes to form an RNA amplification product-detection probe-gold probe complex, which was fixed on the nitrocellulose membrane by lateral chromatography to form a visible band.

### DNA sequencing.

Mutations at sites 2063, 2064, and 2617 in the M. pneumoniae 23S rRNA gene domain V region were detected by direct sequencing of samples with a positive PCR result as described elsewhere (39, 40). Briefly, by mixing primers (Sigma-Aldrich, China), *Taq* polymerase (TaKaRa Bio, Inc., Shiga, Japan), and extracted DNA, nested PCR was performed with a thermal cycler (TaKaRa Bio, Inc., Shiga, Japan). The purified PCR products were labeled with a BigDye Terminator version 3.1 cycle sequencing kit (Applied Biosystems, Foster City, CA, USA) and applied to an ABI Prism 3130xl genetic analyzer (Applied Biosystems, Foster City, CA, USA) in accordance with the manufacturer’s instructions. A sequence scanner (Applied Biosystems, Foster City, CA, USA) was used to determine gene mutations at each site.

### Statistical analysis.

Data are presented as median values and interquartile ranges (IQRs) for continuous variables and frequencies and percentages for categorical variables. Continuous-variable comparisons between the groups were performed using the Mann-Whitney *U* test. Categorical variables were compared using the Fisher exact test. Pearson correlation coefficients were examined to assess whether the prevalence of COVID-19 was associated with the prevalence of M. pneumoniae. Statistical significance was determined as a 2-sided *P* value of <0.05. Statistical analyses were performed using SPSS software 25.0 (IBM SPSS Statistics, Armonk, NY, USA).

### Data availability.

The data used and/or analyzed during the current study were deposited in the Open Archive for Miscellaneous Data (OMIX) under accession no. PRJCA006571 (https://ngdc.cncb.ac.cn/omix/release/OMIX593).
