# Supplementary material for: Mycoplasma pneumoniae among Chinese Outpatient Children with Mild Respiratory Tract Infections during the Coronavirus Disease 2019 Pandemic
Source: Microbiol Spectr. 2022 Feb 9;10(1):e01550-21. doi: 10.1128/spectrum.01550-21 (PMC8826743; doi:10.1128/spectrum.01550-21)
Supplement: SUPPLEMENTAL FILE 1 — Supplemental material. Download SPECTRUM01550-21_Supp_1_seq6.pdf, PDF file, 0.1 MB [file spectrum01550-21_supp_1_seq6.pdf]

Table S1. Canadian acute respiratory illness and flu scale for common cold

Item	No problem (0)	Minor problem (1)	Moderate problem (2)	Major problem (3)	Don't know or Not applicable[a]
Poor appetite					
Not sleeping well					
Irritable, cranky, fussy					
Feels unwell					
Low energy, tired					
Not playing well					
Crying more than usual					
Needing extra care					
Clinginess					
Headache					
Sore throat					
Muscle aches or pains					
Fever					
Cough					
Nasal congestion, runny nose					
Vomiting					
Not interested in what's going on					
Unable to get out of bed					

[a] Score was calculated as the mean of all applicable items.

Table S2 . Cough score for acute tracheobronchitis and post-infection cough

Item	None (0)	Nearly none (1)	Occasionally (2)	Sometimes (3)	Often (4)	Very often (5)	Persistence (6)
**Daytime cough score**							
Frequency							
Severity							
Annoying							
Expectoration							
Wheeze							
**Nocturnal cough score**							
Frequency							
Annoying							
Not sleeping well for the kid							
Not sleeping well for the caregiver							

Table S3. State of the illness

Item	Yes	No
Repeat outpatient visits		
Pneumonia development		
Hospitalization		

Supplement websites

Government websites of Health Commission:

https://wsjkw.sh.gov.cn/;
http://wjw.sz.gov.cn/;
http://jnmhc.jinan.gov.cn/;
http://sxwjw.shaanxi.gov.cn/;
http://wsjk.tj.gov.cn/

Government websites of Bureau of Statistics:

https://tjj.sh.gov.cn/;
http://tjj.sz.gov.cn/;
http://jntj.jinan.gov.cn/;
http://tjj.shaanxi.gov.cn/;
http://stats.tj.gov.cn/
